# 

**Published:** 1847-04

**Authors:** 


### CONTENTS

OF NO. IV.

### ORIGINAL COMMUNICATIONS.

### ESSAYS AND CASES.

ART. I.—An Essay on Eclampsia Parturientium, with Cases and Remarks. By JOHN DAWSON, M.D., of Jamestown, Ohio. - - - - - - - - 277

ART. II.—Notes on Medical Matters and Medical Men in Paris. By DAVID W. YANDELL, M. D., of Louisville, Ky. - - - - - - - - - - 308

ART. III.—A Case of Poisoning by the Seeds of the Datura Stramonium. By Dr. D. G. GREGORY, of Rutersville, Texas. - - - - - - - - - - 333

ART. IV.—A Case of Spontaneous Combustion. By THOS. S. FOSTER, Student of Medicine in the University of Louisville. - - - - - - - - - 334

### BIBLIOGRAPHICAL NOTICES.

ART. V.—Materia Medica and Therapeutics; including the Preparations of the Pharmacopœias of London, Edinburgh, Dublin, and the United States; with many new Medicines. By J. FORBES ROYLE, M.D., F.R.S., Professor of Materia Medica and Therapeutics, King's College, London. Edited by JOSEPH CARSON, M.D., Professor of Materia Medica in the Philadelphia College of Pharmacy. - - - - - - - - - 335

ART. VI.—A Practical Treatise on Inflammation, Ulceration and Induration of the Neck of the Uterus; with Remarks on the Value of Leucorrhœa and Prolapsus Uteri as Symptoms of Uterine Disease. By JAMES

Henry Bennet, M.D., Licentiate of the Royal College of Physicians, etc., Paris. Philadelphia: Lea & Blanchard: 1847. - - - - - - - - 341

Art. VII.—The Principles and Practice of Ophthalmic Medicine and Surgery. By T. Wharton Jones, F.R.S. Edited by Isaac Hays, M.D. Philadelphia. - - 346

Art. VIII.—The Pathological Anatomy of the Human Body. By Julius Vogel, M.D., Professor of Clinical Medicine at the University of Giessen. Translated from the German, with additions, by Geo. E. Day, M.A., etc. Philadelphia: Lea & Blanchard: 1847. - 348

SELECTIONS FROM AMERICAN AND FOREIGN JOURNALS.

Sulphuric Ether—Letheon. - - - - - - - - 349

On the results of Lithrotity Methodically Applied to Suitable Cases. - - - - - - - - - - - 354

Statistics of Miasmatic Fevers treated in 1846, Norfolk, Va. 355

Action of Sulphate of Quinine on the Spleen. - - - - 358

Fever in Ireland. - - - - - - - - - - - 359

Treatment of Aneurism by Compression. - - - - - 359

Child born alive at the fourth month and a half of pregnancy. 359

Treatment of Epistaxis by Insufflations of Alum. - . 359

Death of Dr. Bostock and Dr. John Thompson. - - - 360

ORIGINAL INTELLIGENCE.

Kentucky Institution for the Blind. - - - - - - 361

Examinations for the Doctorate in the University of Virginia. 363

University of Louisville. - - - - - - - - 364

Medical Convention. - - - - - - - - - 364

Bulletin of Medical Science. - - - - - - - - - 365

Resignation of Professor Warren. - - - - - - - 366

Private Medical Instruction in Louisville. - - - - 366

Bloody Vesicle of the Vagina. - - - - - - - - 367

Books Received. - - - - - - - - - - - - 368

